# Effectiveness of attachment-based compassion therapy to reduce psychological distress in university students: a randomised controlled trial protocol

**DOI:** 10.3389/fpsyg.2023.1185445

**Published:** 2023-08-24

**Authors:** María Beltrán-Ruiz, Selene Fernández, Javier García-Campayo, Marta Puebla-Guedea, Yolanda López-del-Hoyo, Mayte Navarro-Gil, Jesus Montero-Marin

**Affiliations:** ^1^Department of Psychology and Sociology, University of Zaragoza, Zaragoza, Spain; ^2^Research Network on Chronicity, Primary Care and Health Promotion (RICAPPS), Zaragoza, Spain; ^3^Department of Psychiatry, University of Zaragoza, Zaragoza, Spain; ^4^Aragonese Institute of Health Research, University of Zaragoza, Zaragoza, Spain; ^5^Department of Psychiatry, Warneford Hospital, University of Oxford, Oxford, United Kingdom; ^6^Teaching, Research & Innovation Unit, Parc Sanitari Sant Joan de Déu, Sant Boi de Llobregat, Spain; ^7^Consortium for Biomedical Research in Epidemiology & Public Health (CIBERESP), Madrid, Spain

**Keywords:** university students, psychological distress, compassion, attachment, mindfulness

## Abstract

**Introduction:**

Higher education, particularly university, is a challenge for many students that can lead to their mental health being seriously affected. The stress to which they are subject throughout their time at university can lead to anxiety and depression. “Third wave” psychotherapies, including compassion-based therapy, have been used to improve psychological outcomes, such as stress, anxiety, emotional distress and well-being. There are some signs that third wave psychotherapies reduce psychological distress in university students, but more and higher-quality studies are needed. In this randomised controlled trial (RCT), we hypothesise that the provision of attachment-based compassion therapy (ABCT) will be more effective than an active control group based on relaxation therapy for improving psychological distress in university students.

**Methods and analysis:**

A two-arm RCT will be conducted involving 140 university undergraduate and postgraduate students from the University of Zaragoza and the National University of Distance Education (UNED) who reside in the autonomous community of Aragon, Spain. Interventions with either ABCT or relaxation therapy will be implemented, with an allocation ratio of 1:1 between groups. Both interventions will last six weeks and consist of six weekly group sessions lasting 1.5 h each. Data will be collected before and after the intervention, and there will be a follow-up at six months. The primary outcome will be psychological distress at post-intervention. Secondary outcomes will be depression, anxiety, stress and burnout symptoms, affectivity and emotional regulation. Attachment style, experiential avoidance, compassion (for others/oneself) and mindfulness skills will be measured as potential mechanistic variables. Intention-to-treat analysis will be performed using linear mixed regression models. The clinical significance of improvements will be calculated. Potential side effects will be monitored by an independent clinical psychologist.

**Ethics and dissemination:**

This study was approved by the Clinical Research Ethics Committee of Aragón. Participant data will remain anonymous, and results will be submitted to peer-reviewed open-access journals and disseminated via conferences.

**Clinical Trial Registration:**

ClinicalTrials.gov, identifier NCT05197595.

## Introduction

### Background and rationale

Recent studies show that university students commonly suffer from mental health problems (Auerbach et al., 2018; Karyotaki et al., 2020). It has been found that more than half of university students might suffer from depression, panic and/or generalised anxiety (Keyes et al., 2012). Anxiety levels increase during university years (Bewick et al., 2010), and the prevalence rates of psychological distress and mental health problems are significantly higher than those of the general population (Stallman, 2010; Jackman et al., 2022). There are numerous areas that can pose a challenge and increase stress for university students, such as financial status, health, love life, family and work/school relationships, and problems experienced by loved ones (Karyotaki et al., 2020). The main mental health pathologies found are depression and anxiety (Regehr et al., 2013), although some university students also report feelings of loneliness, difficulties with family and intimate relationships, and other interpersonal concerns (Conley et al., 2015). All these mental health problems may have psychological and social impacts, and they can also determine students’ ability to function in academic terms, leading to an increase in dropout rates (Dyrbye et al., 2010; Sharp and Theiler, 2018; Marôco et al., 2020), which presents a problem for themselves and for the institutions involved (Conley et al., 2015). Nevertheless, providing psychological tools is believed to be a promising way to enhance students’ resilience and minimise risks of mental health problems (Sharp and Theiler, 2018; Sheldon et al., 2021).

In recent years, there has been an increasing number of studies regarding so-called “third wave” cognitive-behavioural psychological techniques. Through the promotion of acceptance, mindfulness, cognitive defusion and compassion, these therapies seek to change the function of experience for the individual in order to enhance well-being (Hayes et al., 2006). Third wave therapies have demonstrated promising results in different areas related to people’s health and psychological well-being (Grossman et al., 2004; Khoury et al., 2015; Spijkerman et al., 2016; Kirby et al., 2017). A number of studies show that these techniques can reduce anxiety, stress and burnout, and increase psychological well-being in the university population (Regehr et al., 2013; de Vibe et al., 2018; Patel et al., 2018; Dawson et al., 2020). Nonetheless, although their results are promising, some of the studies have also been found to have low methodological quality and considerable heterogeneity of effects (Dawson et al., 2020).

Among third wave psychological techniques, mindfulness-based programmes (MBPs) have predominantly been studied for the prevention and treatment of depressive or anxiety symptoms, and they have also been used in clinical practice for the longest time (Parsons et al., 2017; Cladder-Micus et al., 2018; Goldberg et al., 2018). As examples of how this has materialised, we can see that the National Institute of Health and Care Excellence (NICE) UK guideline recommends an MBP for the prevention of relapses in depression (Kendrick and Pilling, 2012), and that some MBP adaptations for university students have been piloted with positive results (Medlicott et al., 2021). One of the implicit components of MBPs is compassion. However, the effectiveness of compassion-based programmes (CBPs), which use specific practices to develop compassion, is yet to be proven in non-clinical populations (Brito-Pons et al., 2018). A number of pilot studies that include explicit compassion practices and programmes based on compassion therapy have shown encouraging results; however, they were conducted using small samples and therefore require further replications (Arimitsu, 2016; Ko et al., 2018; Collado-Navarro et al., 2021; Martínez-Rubio et al., 2021). In the university population specifically, the efficacy of self-compassion is inconclusive as there is not a great deal of evidence (Dawson et al., 2020) that is consistent with the state of the art in the general population, meaning that further research is required (Kirby et al., 2017; Shonin et al., 2017).

CBPs propose meditative techniques and practices to develop (a) compassion for others, which arises from witnessing the suffering of others and having the desire to alleviate it (Goetz et al., 2010); and (b) self-compassion, which is this same desire but towards oneself (Neff, 2003). The attachment-based compassion therapy (ABCT) programme (García-Campayo et al., 2016; García-Campayo, 2020) seeks to promote compassion for others and self-compassion in individuals through the development of a secure attachment style. Although work on the attachment style has already formed part of some of the compassion programmes on which ABCT is based (Neff, 2003; Gilbert, 2009), ABCT makes the change towards a healthy attachment style the core of the therapeutic process. This programme has obtained satisfactory results by increasing self-compassion in healthy adults (Navarro-Gil et al., 2020) and reducing affective distress in patients with anxiety, depressive and adjustment disorders (Collado-Navarro et al., 2021), and its clinical usefulness with fibromyalgia patients has also been demonstrated (Montero-Marin et al., 2019; Santos et al., 2022), with results maintained in the medium term.

This present study will continue this line of research by assessing the effectiveness of an adapted six-week ABCT programme in the treatment of psychological distress in university students, compared with relaxation therapy. It will also evaluate the potential mediating role of attachment style, experiential avoidance [i.e., the unwillingness to experience painful thoughts and emotions (Berta-Otero et al., 2022)], compassion (for others/oneself) and mindfulness skills (i.e., an awareness of the present moment that is characterised by a non-judgmental attitude) in the intervention group, compared to the active control group based on relaxation therapy. There is some preliminary evidence to show that experiential avoidance, self-compassion and mindfulness skills having a potential mediating role in ABCT (Montero-Marin et al., 2019; Collado-Navarro et al., 2021; Lopez-del-Hoyo et al., 2022). However, there are no previous studies evaluating attachment style and compassion for others as putative mechanisms of ABCT, even though they are core constructs of the programme.

### Objectives and hypotheses

The main aim of this study will be to assess the effectiveness of a six-week ABCT programme, compared with an active control group based on relaxation therapy (including progressive muscular relaxation and guided imagery), for the reduction of psychological distress symptoms in university students. Secondary objectives are (a) to examine the effects of ABCT on anxiety, depression, stress and burnout symptoms, as well as positive and negative affect, and emotional regulation and (b) to analyse the possible mediating role of attachment style, experiential avoidance, compassion (for others/oneself) and mindfulness skills on improvements in the ABCT group, compared to the control group based on relaxation therapy.

The main hypothesis is that ABCT will be more effective than relaxation therapy to reduce psychological distress in university students at post-treatment. Secondary hypotheses are the following: (a) ABCT will be more effective than relaxation therapy for the improvement of anxiety, depression, stress and burnout symptoms, as well as positive and negative affect, and emotional regulation at post-treatment; (b) improvements in psychological distress, anxiety, depression, stress, burnout, positive and negative affect, and emotional regulation will be maintained at six-month follow-up; and (c) attachment style, experiential avoidance, compassion (for others/oneself) and mindfulness skills will have a mediating role on the improvements obtained in the ABCT group vs. the control group based on relaxation therapy.

### Trial design

This study will be a randomised controlled trial (RCT) comprising two parallel arms, with pre-treatment, post-treatment and six-month follow-up measurements, and a 1:1 allocation ratio between groups. University students in Zaragoza, Spain, will be randomly assigned to two different conditions: ABCT (intervention group) and relaxation therapy (active control).

## Methods

This protocol was designed in accordance with the Standard Protocol Items: Recommendations for Interventional Trials (SPIRIT) statement (Moher and Chan, 2014). The trial registration can be found at ClinicalTrials.gov NCT05197595 (January 19, 2022).

### Setting and study sample

We will recruit students from the University of Zaragoza and the National Distance Education University (UNED), both in Spain, who meet the following inclusion/exclusion criteria. Inclusion criteria: (a) over 17 years of age; (b) studying for an undergraduate or postgraduate (master’s or PhD) degree; (c) proficient in spoken and written Spanish; and (d) provide signed informed consent. Exclusion criteria: (a) over 30 years of age; (b) diagnosed with a disease that affects the Central Nervous System or a serious mental illness; and (c) consumption of recreational drugs or on medication that could affect the nervous system. Concomitant care will be permitted during the trial if treatment has been initiated previously and consists of a maintenance dose (no increments or psychological interventions will be allowed).

### Sample size

The sample size estimation was calculated based on the assumption that the ABCT group would be able to obtain intermediate effects when compared to the active control condition based on relaxation therapy. In order to determine this, we considered a standardised difference between trial arms on the main outcome (psychological distress, measured by the Depression, Anxiety and Stress Scale (DASS-21) total scale) of 0.5, which is the more conservative estimation observed in a previous similar study that included university students and delivered a third wave psychological intervention based on six meditation sessions (Modrego-Alarcon et al., 2021), and is usually considered a common rule of thumb of clinically significant change (Norman et al., 2003). Considering a power (1-β) of 0.80 for a two-tailed contrast and an α of 0.05 with a group allocation ratio of 1:1, we obtained a group size of approximately 64 subjects. Therefore, the total size of the required sample will be 128 voluntary participants from the university student population. Assuming a loss rate of around 30%, based on a previous similar study (Modrego-Alarcon et al., 2021), we have inflated the numbers so that the total sample size needed will be approximately 190 subjects, 95 in each group.

### Recruitment

Recruitment of potential participants will begin as follows: (1) informative posters providing a brief explanation of the study will be displayed around the main campus of the University of Zaragoza at tutoring whose services are used by students of distance courses (for UNED students) and other places of interest for university students to encourage interested students to make contact via e-mail; (2) contact will be made with different student organisations to send informative e-mails to students. Each organization will send the message using e-mail lists, without providing the research group with any personal information; and (3) contact with local government agencies and media (e.g., newspapers) to disseminate information about the research study and provide a contact e-mail for any interested students.

Those students who make e-mail contact will be provided with more detailed information by means of a phone call or virtual meeting. A research assistant will explain the study and verify that the student meets the inclusion criteria. This contact will also specify the route by which the study information document is to be received, and a link will be provided to the baseline evaluation. All assessments which will be made online, both at baseline and in the subsequent follow-up measurements at the end of the intervention (post-treatment), and at six months, and the surveys will be completed online using SurveyMonkey^®^. Before beginning the baseline survey, the participants will be able to read the information sheet, accept the privacy terms and conditions of the website, and sign the informed consent form. The recruitment and baseline assessment process will continue until the required sample size is obtained.

### Randomisation, allocation, and blinding

Once baseline data is collected, participants will be randomly allocated to each arm. The random assignment of the subjects will be carried out by a different member of the research group who is unrelated to the study and using a computer-generated random sequence. This sequence will be blinded to both the participants and the research assistants who will be assisting with the assessments (i.e., by providing the link to the survey on the appropriate date), as well as to the trial manager who will be in charge of group allocation. Participants will be assigned to one of two groups: ABCT or relaxation therapy. Given the nature of the interventions, neither the group facilitators (i.e., psychotherapists) nor the university student participants will be blinded to their allocation. However, the research assistant in charge of providing the link to the assessment survey will be blinded, as well as the expert who will carry out the statistical analysis.

### Data collection and monitoring

**Figure 1:**
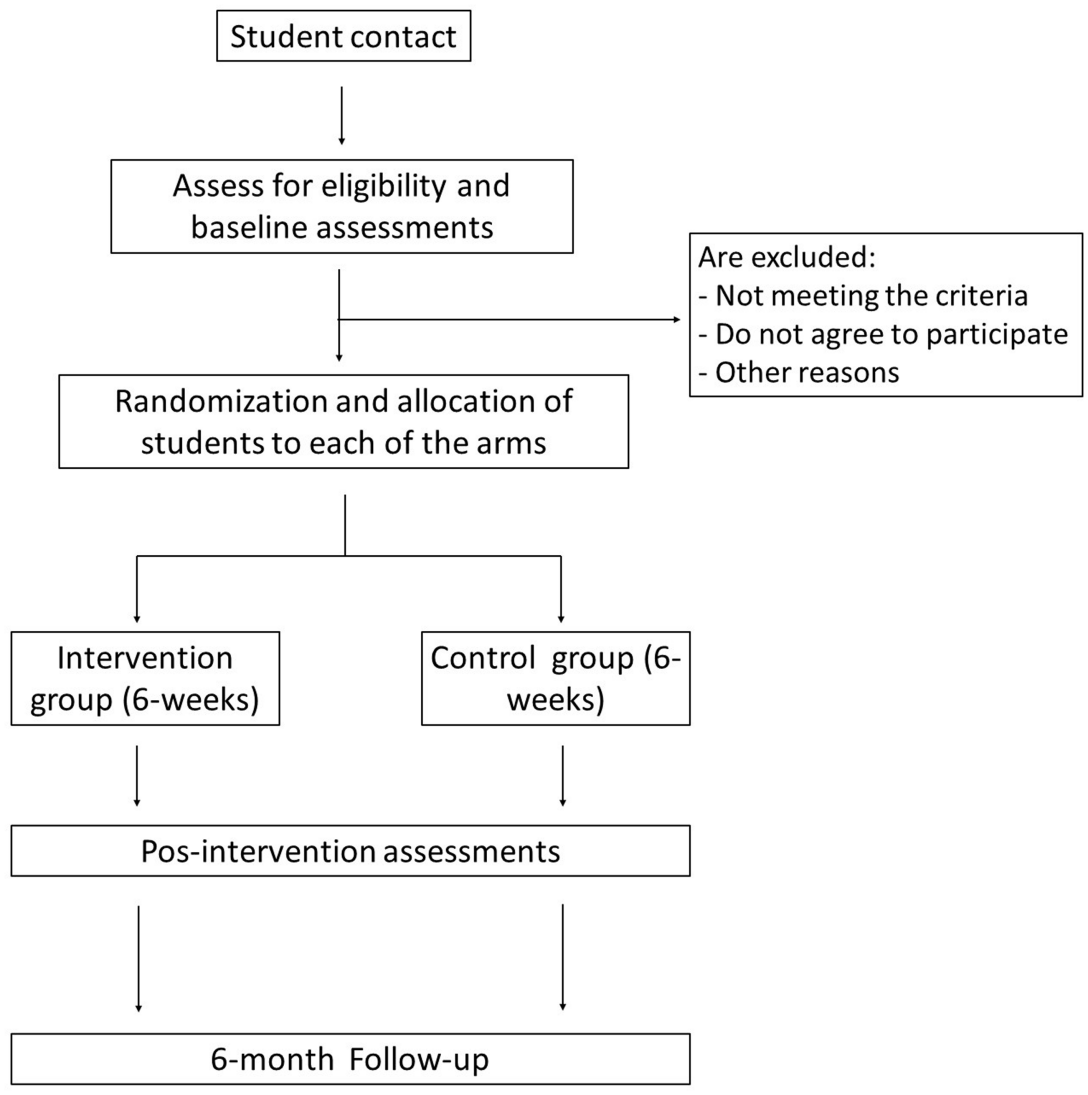
Study flowchart.

After completing the programmes, the same person who provided the links to complete the baseline survey will oversee the process of contacting the participants so that they can carry out the post-test (with a time window of one month after the intervention), and follow-up (six months after the last session of the programme, with a time window of one month) measurements. Participation in meditation programmes, as in the case of any psychotherapeutic approach, can cause negative side effects that, although rare, need to be monitored (Van Dam et al., 2018; Baer et al., 2021). Therefore, study participants will be asked to report any signs of a worsening in their mental or physical health (whether serious or not) that may arise during the sessions. Participants will also be asked to comment on any discomfort they experience both during and between sessions. If adverse effects appear, the psychologist in charge of the groups will discuss with an independent data monitoring committee (which will be made up of the trial manager, a clinical psychologist and a psychiatrist) whether any additional measures need to be taken to ensure the integrity of the participant (e.g., abandon the study). The results of the surveys at each time point will also be overseen by the data monitoring committee, who will decide whether any potential deterioration of psychological health needs further consideration or treatment. This will be facilitated by contacting the corresponding mental health services of national health system. Figure 1 is the flow diagram that shows the expected participation of the subjects throughout the study, from recruitment to follow-up data collection.

### Interventions

Both groups will receive interventions with a duration of six weeks. Each session will be 90 min long and will be held in face-to-face groups. Before beginning the programme, participants will be given a paper copy of the information sheet and the informed consent form, and they will be able to raise any doubts they might have regarding the study.

#### Attachment-based compassion therapy

The ABCT programme that will be used is a short adaptation of the University of Zaragoza compassion training programme (García-Campayo et al., 2016). Table 1 shows how the sessions are structured in terms of theory as well as formal and informal practices. Tasks to consist of practical exercises using audio recordings will be set for completion between sessions. The ABCT programme will be conducted by a clinical psychologist with experience in ABCT and third generation therapies.

**Table 1:** Adaptation of the ABCT programme (6 weeks).

Session 1	Preparing for compassion: kind attention.	Theory: the functioning of our brain, the reality of suffering: primary and secondary suffering, what is and what is not compassion. Formal practices: compassionate breathing and compassionate body scan, compassionate coping with difficulties. Informal practices: 3-Minute compassion practice, self-compassion journal, savour and thank you
Session 2	Discovering our compassionate world	Theory: compassion and mindfulness, self-esteem and compassion, fear of compassion. Formal practices: connect with affection, develop a situation or a safe place, compassionate gesture and phrases. Informal practices: the object that unites us to the world, compassion practice diary, what are we good at?
Session 3	Developing our compassionate world	Theory: mechanisms of action of compassion, efficacy of compassion, self-criticism. Formal practices: identify and develop the secure attachment figure, replace the critical voice with the compassionate voice. Informal practices: write a letter to the attachment figure.
Session 4	Understanding our relationship with compassion	Theory: attachment models, importance of these models in daily life. Formal practices: becoming aware of our attachment model, receiving affection from friends, indifferent and enemies. Informal practices: Letter to parents
Session 5	Working on ourselves	Theory: importance of affection towards oneself and towards others Formal practices: giving affection to friends and indifferent, giving affection to ourselves, reconciliation with our parents. Informal practices: three positive and three negative aspects of our parents, the largest sample of affection (in general and from our parents)
Session 6	Understanding the importance of forgiveness	Theory: guilt and the importance of forgiveness Formal practices: become aware of the damage we have done to others and ask for forgiveness, forgive others, and give compassion to enemies, forgive oneself. Informal practices: compassion in daily life

#### Relaxation therapy

The progressive muscle relaxation programme proposed by Bernstein and Borkovec (1973) will be used. To match this programme to the ABCT programme, it will be adapted to six sessions, each lasting 90 min, with the addition of guided imagery. As in the previous case, tasks to consist of practical exercises using audio recordings will be also set for completion between sessions. The contents and structure of the sessions are described in Table 2. The relaxation program will be facilitated by a clinical psychologist with experience in relaxation techniques.

**Table 2:** Relaxation programme (6 weeks).

Session 1	Presentation of the group and the goals of relaxation Basic principles of progressive muscle relaxation Brief explanation of the initial procedure with 16 muscle groups Progressive muscle relaxation practice with 16 muscle groups Imagination training Identification of the sensations and difficulties of relaxation
Session 2	Share homework related experiences and doubts Theory of visualisation techniques Practice of progressive muscle relaxation with 16 muscle groups Visualisation Practice: The Orange Identification of sensations and difficulties of relaxation
Session 3	Share homework related experiences and doubts Brief explanation of the procedure with 7 muscle groups Practice of progressive muscle relaxation with 7 muscle groups Visualisation Practice: The Beach Identification of sensations and difficulties of relaxation
Session 4	Share homework related experiences and doubts Practice of progressive muscle relaxation with 7 muscle groups Visualisation practice: the landscape Identification of sensations and difficulties of relaxation
Session 5	Share homework related experiences and doubts Brief explanation of the procedure with 4 muscle groups Practice of progressive muscle relaxation with 4 muscle groups Visualisation practices: the globe and white light Identification of sensations and difficulties of relaxation
Session 6	Share homework related experiences and doubts Brief explanation of relaxation by evocation, relaxation by evocation + counting and relaxation by counting Relaxation by evocation + counting Relaxation by counting Mental relaxation and visualisation practice: the perfect day Identification of sensations and difficulties of relaxation

### Outcomes

Data will be collected using a battery of questionnaires administered at baseline, immediately after the intervention (post-treatment) and at six-month follow-up. Outcomes of the ABCT and the control conditions will be evaluated and compared. The primary outcome will be a self-reported global measure of psychological distress to provide data on the intervention. Secondary outcomes will allow a more detailed exploration of ABCT in terms of depression, anxiety, stress, burnout, emotional regulation and affectivity. Furthermore, the role of attachment style, experiential avoidance, compassion (for others/oneself) and mindfulness skills as potential mediators of outcome will be explored. A breakdown of the study outcomes is given in Table 3.

**Table 3:** Study outcomes.

Instrument	Assessment area	Kind of outcome	Time
Bespoke survey	Sociodemographic	Characteristics of participants	Baseline
DASS-21	Psychological distress	Primary (total score) Secondary (sub-scales) outcome	Baseline, post-treatment and 6-month follow-up
PANAS	Positive and negative affect	Secondary outcome	Baseline, post-treatment and 6-month follow-up
ERQ	Emotional regulation	Secondary outcome	Baseline, post-treatment and 6-month follow-up
MBI-SS	Burnout	Secondary outcome	Baseline, post-treatment and 6-month follow-up
RQ	Attachment style	Mechanistic measures	Baseline, post-treatment and 6-month follow-up
AAQ-II	Experiential avoidance	Mechanistic measures	Baseline, post-treatment and 6-month follow-up
SOCS-O	Compassion for others	Mechanistic measures	Baseline, post-treatment and 6-month follow-up
SOCS-S	Compassion for the self	Mechanistic measures	Baseline, post-treatment and 6-month follow-up
FFMQ-SF	Mindfulness	Mechanistic measures	Baseline, post-treatment and 6-month follow-up

DASS-21, depression, anxiety and stress scales, 21 items; PANAS, positive and negative affect schedule; ERQ, emotional regulation questionnaire; MBI-SS, Maslach burnout inventory student survey; RQ, relationship questionnaire; AAQ-II, acceptance and action questionnaire-II; SOCS-O, Sussex-Oxford compassion for others scale; SOCS-S, Sussex-Oxford compassion for the self scale; FFMQ-SF, Five facet mindfulness questionnaire short-form.

#### Main outcome

The main effectiveness outcome will be psychological distress at post-intervention as the primary endpoint, which will be assessed using the short version of the Depression, Anxiety and Stress Scales (DASS-21) (Lovibond and Lovibond, 1996). The DASS-21 is a self-report questionnaire composed of the three negative affectivity subscales of depression, anxiety and stress. Each of the subscales comprises seven items with Likert-type response options (from 0 “did not apply to me at all” to 3 “applied to me very much, or most of the time”). The result of each of these scales will be doubled to achieve equivalence with the long version of 42 items (Lovibond and Lovibond, 1996). The DASS-21 has been specifically validated in the Spanish population, showing strong internal consistency values (total scale *α* = 0.96; depression *α* = 0.93; anxiety *α* = 0.86, and stress *α* = 0.91), as well as appropriate patterns of discriminant, convergent and factorial validity (Daza et al., 2002). The DASS-21 total scale score will be considered the main outcome, and the DASS-21 subscales (i.e., depression, anxiety, and stress) will be considered secondary outcomes. To facilitate interpretation, and following Chin et al. (2019), we will also use a secondary pre-defined binary outcome measure based on the DASS-21 total score that will differentiate those participants who scores 16 points or more to identify individuals with potential anxiety disorders or a major depressive disorder.

#### Secondary outcomes

Burnout symptoms will be evaluated using the Maslach Burnout Inventory Student Survey (MBI-SS) (Schaufeli et al., 2002). This inventory consists of 15 items, in which references to work are changed to references to study. The MBI-SS includes three subscales: exhaustion (5 items), cynicism (4 items) and efficacy (6 items). Participants respond on a Likert-type scale with seven response options ranging from 0 (“never”) to 6 (“always”). The psychometric properties of the MBI-SS Spanish validation have been observed to be adequate (exhaustion *α* = 0.83, cynicism *α* = 0.83 and efficacy *α* = 0.82) (Pérez Fuentes et al., 2020).

Positive affect and negative affect will be evaluated by means of the Positive and Negative Affect Schedule (PANAS) (Watson et al., 1988). This self-report questionnaire consists of two 10-item scales (i.e., positive affect, and negative affect). Each item is scored on a five-point Likert-type scale, from 1 (“not at all”) to 5 (“very much”). The internal consistency of the Spanish version of the PANAS positive and negative scales is adequate, with values of *α* = 0.87 and 0.91, respectively (Sandín et al., 1999).

Emotional regulation will be measured by the Emotional Regulation Questionnaire (ERQ) (Gross and John, 2003). This scale consists of 10 items, to which participants respond using a seven-point Likert scale (1 = “strongly disagree”, 7 = “strongly agree”). The ERQ is designed to measure the tendency of respondents to regulate their emotions through (1) cognitive reappraisal (6 items), and (2) expressive suppression (4 items). The Spanish version of the ERQ shows an adequate internal consistency (cognitive reappraisal: *α* = 0.89–0.90; expressive suppression: *α* = 0.76–0.80), test–retest reliability and convergent/discriminant validity (Cabello et al., 2013).

#### Mechanistic measures

Attachment style will be measured using the Relationship Questionnaire (RQ) (Bartholomew and Horowitz, 1991), a self-report questionnaire in which participants are asked to rate their correspondence to four separate paragraphs, each representing a secure, preoccupied, dismissive or fearful attachment style, by means of a seven-point Likert-type scale. An algorithm allows for a categorical classification of attachment style (i.e., secure, or insecure) (Griffin and Bartholomew, 1994a). The RQ also offers the possibility of measuring two key dimensions underlying attachment in adults: anxiety, which is more self-related, and avoidance, which is more other-related (Griffin and Bartholomew, 1994b). Studies have demonstrated reliability of the RQ questionnaire to be high (Leak and Parsons, 2001). The validated Spanish version of the RQ, which shows adequate psychometric properties (Yárnoz-Yaben and Comino, 2011), will be used in our study.

Experiential avoidance will be assessed with the Acceptance and Action Questionnaire-II (AAQ-II) (Bond et al., 2011). The AAQ-II is a measure of experiential avoidance as an aspect of lack of psychological flexibility. It is made up of seven items on a seven-point Likert-type scale, where 1 is “never true” and 7 is “always true”. The items reflect a lack of willingness to experience unwanted emotions or thoughts, and a lack of ability to be in the present moment and behave according to what is valued when experiencing unwanted psychological events. The instrument presents a unifactorial solution, with good internal consistency (*α* = 0.88), and good convergent, divergent and discriminant validity (Berta-Otero et al., 2022). An adaptation to Spanish will be used (Ruiz et al., 2013).

Compassion will be assessed using the Sussex-Oxford Compassion for Others Scale (SOCS-O) and the Sussex-Oxford Compassion for the Self Scale (SOCS-S) (Gu et al., 2020). The SOCS-O and SOCS-S represent two dimensions, compassion for others and self-compassion, respectively, with 20 items each. Participants indicate how true each statement is using a five-point Likert-type scale, ranging from 1 (“not entirely true for me”) to 5 (“always true for me”). A total score is calculated for the SOCS-O and for the SOCS-S, with higher scores meaning greater levels of compassion for others or self-compassion. The study will use an adaptation of the scale to the Spanish language, which is currently being validated ^1^

Mindfulness skills will be measured with a short version (24 items) of the Five Facets of Mindfulness Questionnaire (FFMQ-SF) (Bohlmeijer et al., 2011). The FFMQ questionnaire is grouped into five mindfulness facets: observing, describing, acting with awareness, non-judging of inner experience, and non-reactivity to inner experience. Participants must indicate the degree to which each of the items is generally true for them on a five-point Likert-type scale, from 1 (“never or very rarely true”) to 5 (“very often or always true”). Scores from the subscales can be combined to produce a total score. The Spanish version of FFMQ-SF presents good internal consistency values for the total score (*α* = 0.70) and subscales (*α* values ranging from 0.65 to 0.80), and an appropriate factorial structure (Cebolla et al., 2012; Asensio-Martínez et al., 2019).

## Data analysis plan

The results will be presented in accordance with CONSORT recommendations (Moher et al., 2001; Moher and Chan, 2014). All the variables will be described and subject to visual inspection at baseline by using frequencies (proportions) for qualitative variables, or means (standard deviations, SD) for quantitative variables.

### Main analysis

The effectiveness of the ABCT group vs. the control group based on relaxation therapy will be evaluated at post-treatment on the main DASS-21 total score, considered as a continuous variable. Multilevel mixed effects linear regressions will be carried out by means of a repeated measures design on an intention-to-treat (ITT) basis, using the restricted maximum likelihood (REML) method. Non-standardised slopes and 95% confidence intervals (95% CIs) for the “group × time” interaction will be provided, together with raw means (SDs) by group. Cohen’s *d* effect size (ES) will be calculated using the combined SD at baseline (Morris, 2008). ESs are considered small when *d* ≤ 0.2; medium when *d* = 0.5; and large when *d* ≥ 0.8 (Cohen, 1988).

### Secondary analysis

The effectiveness of the ABCT group vs. the relaxation group regarding the secondary outcomes, as well as secondary time points, will be evaluated following the same analytical strategy used for the main analysis. Per protocol analysis will also be performed, considering only those university student participants who attend at least three sessions (out of six). The clinical significance of improvements between groups will be explored by calculating the absolute risk reduction and number needed to treat (NNT) (and their 95% CI) for the DASS-21 total scores. We will use three criteria for improvement: (i) changing to a less severe cluster in the DASS-21 total score, compared to the one the patient was allocated to at baseline (Chin et al., 2019); (ii) calculating reliable change; and (iii) the clinically significant change of improvements by establishing both reliable change and the cut-off point on the DASS-21 total score, using the Jacobson and Truax method (Jacobson and Truax, 1992).

### Mediation analysis

The potential mediating role of the proposed mechanistic variables will be explored in both the primary and secondary outcomes. For this purpose: (i) primary and secondary outcome pre-follow-up differential scores will be calculated and considered dependent variables; (ii) pre-post differential scores of attachment style, experiential avoidance, compassion (others/self) and mindfulness skills will be calculated and included as potential mediators; and (iii) the group condition (ABCT vs. relaxation therapy) will be considered the independent variable. Indirect effects (IEs) will be estimated using path analyses. Regression coefficients of bootstrapped IEs will be calculated, as well as their 95% CIs based on 10,000 bootstrap samples, considering a significant mediating effect when the mentioned bootstrapped 95% CI does not include zero (Lockhart et al., 2011). The percentage of the mediating effects will also be calculated.

### Level of significance

An alpha level of 0.05 will be established using a two-tailed test.

## Discussion

Different studies conducted over the years have pointed out that the mental health of university students is being increasingly compromised (Storrie et al., 2010; Macaskill, 2013). The university population faces a difficult period with many stressors that can trigger a number of serious mental disorders (Karyotaki et al., 2020). Psychological distress has been identified as one of the most important points to be improved in the mental health of young university students (Williams et al., 2015). As a result of all of this, interventions that aim to improve mental health in university students have been widely used (Regehr et al., 2013). Specifically, it has been proposed that programmes aimed at reducing stress and improving well-being among university students should include experiential avoidance, self-compassion and mindfulness skills as therapeutic targets (Martinez-Rubio et al., 2023). In fact, some interventions based on acceptance, compassion and mindfulness training have shown positive results in reducing psychological distress symptoms (de Vibe et al., 2018; Patel et al., 2018; Dawson et al., 2020). CBPs propose practices through which to develop compassion for others and self-compassion (Goetz et al., 2010), and in the case of ABTC, the use of attachment style is proposed as the common thread to generate compassion (for others/oneself) by building a secure attachment figure (García-Campayo et al., 2016; García-Campayo, 2020). ABCT has been shown to be effective in reducing psychological distress in both clinical and non-clinical populations. Some studies show promising results of the use of CBPs in university students (Arimitsu, 2016; Ko et al., 2018; Collado-Navarro et al., 2021; Martínez-Rubio et al., 2021).

With regard to the strengths of this study, although compassion-based approaches show promise for treating the mental health of university students, this study will be one of the first RCTs to evaluate the efficacy of CBPs on university students’ mental health, and the first to verify the specific potential benefits of ABCT for this population. In addition, the study will make use of a randomised controlled design with a relatively large sample of university students and a medium-term (6 months) follow-up, as recommended by previous research on this population (Dawson et al., 2020). This will allow changes to be observed in the medium term and mediation analyses to be carried out. It is also important to point out that an active control group will be used, as recommended in research using contemplative programmes (Ma and Teasdale, 2004), since it allows possible changes in the variables to be attributed to the intervention and not to other factors, such as the attention shown by the instructor or the relationship with the group.

As limitations, we need to recognise that the use of self-report measures can be biased due to social desirability. Another possible barrier is the fact that these programmes require consistent home-based practice between sessions (Grossman et al., 2004). This can lead to the distraction of part of the sample due to fatigue or lack of commitment to the programme.

## Ethics statement

The studies involving human participants were reviewed and approved by Clinical Research Ethics Committee of Aragón (registration: PI21-395). The patients/participants provided their written informed consent to participate in this study.

## Author contributions

MB-R, JG-C, and JM-M conceptualised and designed the study. MB-R and JM-M wrote the first draft of the protocol. JM-M developed the statistical analysis plan. SF, JG-C, MP-G, YL-d-H, and MN-G reviewed the manuscript content. All authors contributed to the article and approved the submitted version.

## Funding

The project has received funding from the DGA Mental Health Research group (B17_23R), from the Mental Health in Primary Care research group dependent on the Aragonese Institute of Health Research (GIIS017), and from the Chronicity, Primary Care and Health Research Network. Health Promotion (RICAPPS) RD21/0016/ Grant 0005 from the Carlos III Health Institute of the Spanish Ministry of Economy and Competitiveness, co-financed with FEDER funds from the European Union. The funders have no role in study design, data collection and analysis, publication decision, or manuscript preparation. JM-M has a Miguel Servet contract from the Institute of Health Carlos III (ISCIII; CP21/00080).

## Conflict of interest

The authors declare that the research was conducted in the absence of any commercial or financial relationships that could be construed as a potential conflict of interest.

## Publisher’s note

All claims expressed in this article are solely those of the authors and do not necessarily represent those of their affiliated organizations, or those of the publisher, the editors and the reviewers. Any product that may be evaluated in this article, or claim that may be made by its manufacturer, is not guaranteed or endorsed by the publisher.

## Footnotes

^1^ Garcia-Campayo, J., Barcelo-Soler, A., Martinez-Rubio, D., Navarrete, J., Perez-Aranda, A., Feliu-Soler, A., et al. (under review). Exploring the relationship between self-compassion and compassion for others: the role of psychological distress and wellbeing.
